# Prevalence and risk factors of subjective cognitive decline in older adults in Baotou, China: a cross-sectional study

**DOI:** 10.3389/fnagi.2024.1422258

**Published:** 2024-10-09

**Authors:** Shang-Jia Ma, Yan-Xue Yu, Kai Tian, Wen Yong, Wen-Long Yu, Ru-Yu Bai, Li-E Wu, Xia Guo

**Affiliations:** ^1^Department of Neurology, The First Affiliated Hospital of Baotou Medical College, Baotou, China; ^2^Department of Neurological Function, Luoyang Central Hospital, Luoyang, China; ^3^Department of Psychological Rehabilitation, The Third Hospital of Baogang Group, Baotou, China; ^4^Department of Neurology, The Ninth People’s Hospital of Shenyang, Shenyang, China

**Keywords:** subjective cognitive decline, prevalence, risk factors, gender, a cross-sectional study

## Abstract

**Objectives:**

Subjective cognitive decline (SCD) as a stage between healthy cognition and early neurocognitive disorders, has been proposed to be helpful in the diagnosis of prodromal neurocognitive disorders. To investigate the prevalence of SCD and the related risk factors on the prevalence.

**Methods:**

A cross-sectional study involving 1,120 elderly subjects residing in Baotou, China. From June 2021 to June 2023, the data were gathered by research assistants with training utilizing standardized questionnaires. The following factors were evaluated: subjective cognitive decline, physical and cognitive activity levels, past medical history, demographics, instrumental activities of daily living, and cognitive function. Risk factors of SCD were used chi-square tests and multivariate logistic regression analysis.

**Results:**

The prevalence of SCD was 43.8%. Permanent residence, marital status, BMI, dietary habits, average sleep duration per night, smoking, diabetes, coronary heart disease, and visual impairment were significantly associated with SCD (*p* < 0 0.05). Multivariable logistic regression analysis showed obesity, vegetarian-based, smoking for a long time, diabetes and coronary heart disease, visual impairment, no spouse, and average sleep duration per night <6 h were independent risk factors for SCD. Based on the gender analysis, the difference in marital status, dietary habits, average sleep duration per night, smoking, drinking, and hypertension was statistically significant (*p* < 0.001).

**Conclusion:**

The prevalence of subjective cognitive decline was high among elder adults. We discovered significant differences in the prevalence or risk factors for SCD between men and women based on their sex. This study provides a more theoretical basis for the early prevention and screening of cognitive impairment diseases in the elderly population.

## 1 Introduction

As a long-term neurodegenerative condition, Alzheimer’s disease (AD) cannot be cured. By 2050, it is predicted that 100 million individuals will suffer from AD dementia globally (Palmqvist et al., 2020). Patients, their families, and society all bear a heavy load of suffering due to dementia (Ren et al., 2022). For patients, it causes more comorbid conditions and increases reliance. High burden and psychological morbidity rates, as well as social isolation, physical illness, and financial difficulty, are prevalent among family caregivers (Jia et al., 2018). Thus, achieving “early detection, diagnosis, and treatment” is crucial. To improve the therapeutic window for Alzheimer’s disease (AD), efforts have focused on identifying individuals when they are in the earliest stage of AD (Chapman et al., 2021).

Subjective cognitive decline (SCD) refers to the self-experience of reduced cognitive functioning despite no signs of objective cognitive impairment from a neuropsychological performance or daily functioning assessment (Jessen et al., 2014). SCD is a key requirement for diagnosing mild cognitive impairment (MCI) and prodromal dementia, even though it may exist without any obvious signs of objective cognitive impairment (Molinuevo et al., 2017). SCD, as a stage between healthy cognition and early neurocognitive disorders, has been proposed to be helpful in the diagnosis of prodromal neurocognitive disorders (Jack et al., 2018). 14% of people with SCD will eventually acquire dementia, and 27% will eventually advance to MCI (Mitchell et al., 2014). Thus, understanding the early pathogenic processes of AD and detecting the early detection of AD requires research into populations with SCD (Rabin et al., 2015).

Studies have shown that the prevalence of SCD varies depending on the survey population, evaluation method, and criteria (Jessen et al., 2020; Pike et al., 2022). European surveys show that about 50% of German adults are concerned about their memory (Luck et al., 2018); the prevalence of SCD in Sweden is 8.96 to 58.1% (Garcia-Ptacek et al., 2016), and in Greece is about 84.20% in people over 65 years old (Vlachos et al., 2019). A Centers for Disease Control and Prevention (CDC) analysis of data from 22 states in the 2015–2019 Behavioral Risk Factor Surveillance System (BRFSS) found that approximately 11% of people aged 45 years and older had SCD, and the prevalence varied slightly by state 9.8–17.3% (Jeffers et al., 2021). The prevalence of SCD has been reported in 17.4% of middle-aged (<65 years old) and 29.4% of older adults (≥ 65 years old) among community-dwelling older adults in Korea (Roh et al., 2021). In a study in Shunyi District, Beijing, the prevalence of SCD between the ages of 60 and 80 was 14.4–18.8% (Hao et al., 2017). In community studies in Zhaoyuan and Guangzhou, the prevalence of SCD in people aged 60 years and older was 32.2 and 58.4%, respectively (Lin et al., 2022; Luo et al., 2024).

The bio-psycho-social model integrates data on biological, psychological, and social components to attempt to provide a comprehensive view of medical research in order to explain health and disease (Engel, 1977; Korbmacher et al., 2023). Current research shows links between brain age and bio-psycho-social factors, such as lifestyle, biomedical, behavioral, and cognitive aspects (Leonardsen et al., 2022; Sone et al., 2022). This study aimed to determine the prevalence of SCD and associated factors in older adults over 60 years of age in the Baotou area based on the bio-psycho-social model, which will benefit health systems to focus on higher-risk groups and intervening cognitive decline in older adults.

## 2 Methods

### 2.1 Study design and participants

This cross-sectional study enrolled subjects aged 60 or older in the Baotou region of Inner Mongolia of China, selected by cluster sampling between June 2021 and June 2023, and a multi-stage stratified cluster sampling design was used to select the participants.

The inclusion criteria was permanent residents in Baotou for at least 6 months. The exclusion standards were (1) neurological conditions such as Parkinson’s disease, encephalitis, brain tumors, brain trauma, cerebrovascular illness, and others that may cause cognitive impairment; (2) metabolic conditions, such as anemia, thyroid dysfunction, a deficiency in folic acid, and a lack of vitamin B12; (3) a history of CO poisoning; (4) a history of general anesthesia; (5) dementia; (6) an acute or serious life-threatening condition; (7) severe vision, hearing, or speech difficulties; and (8) being unable to participate in the neuropsychological evaluation or absence of willingness to participate in the survey freely expressed in the informed consent.

**Figure 1:**
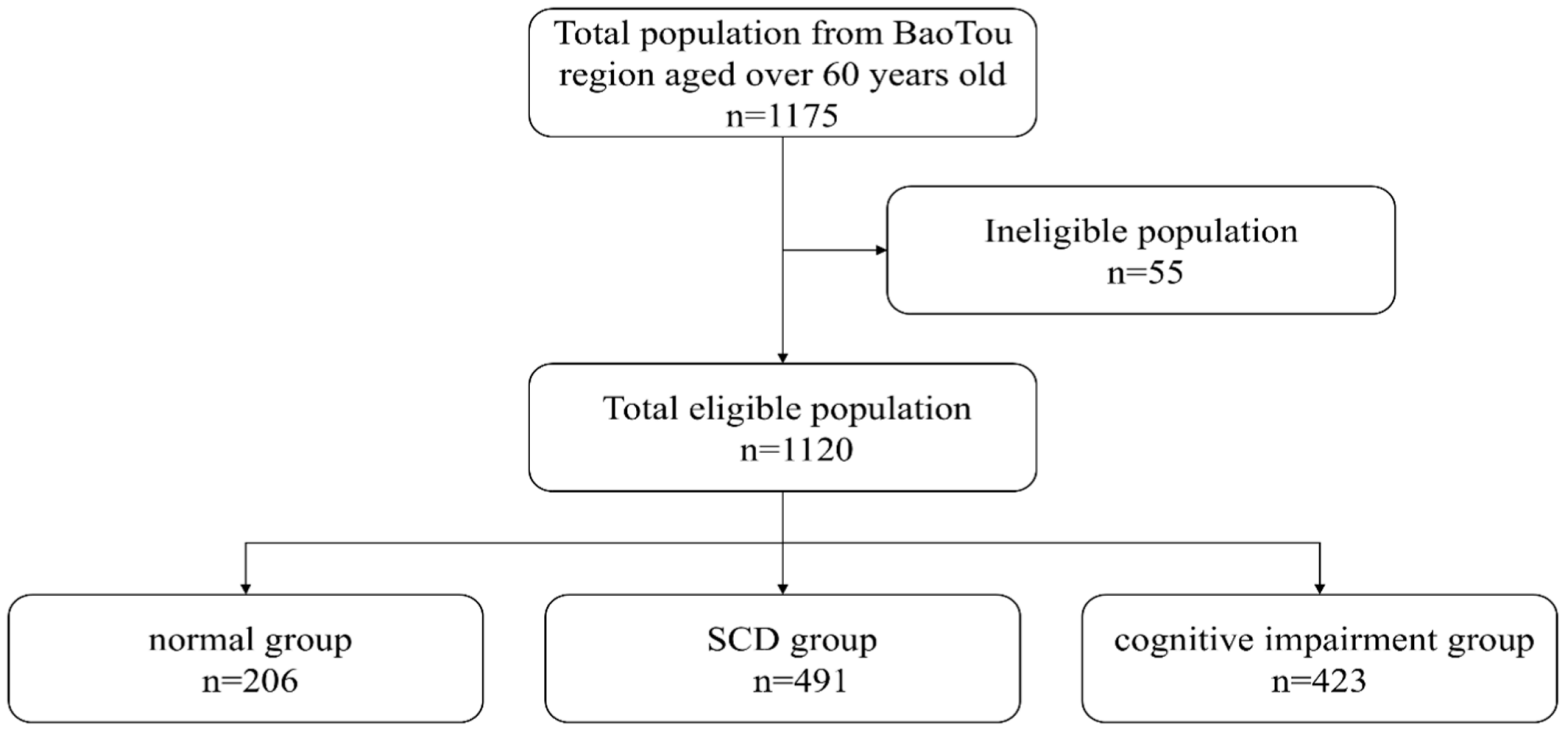
Flow chart.

A random sample of the Baotou region was used to choose the participants. Included were permanent residents older than 60 who had not yet received a dementia diagnosis. According to the maximum sample size needed was determined Based on the calculation equation


$$n=\frac{z_{a}^{2}*p*\left(1-p\right)}{\delta^{2}}$$

and the 10% missed follow-up rate. One thousand one hundred seventy-five study respondents were included, and 1,120 completed this survey with a 95.3% response rate (Figure 1).

### 2.2 Date collection

The questionnaire consisted of general information and cognitive assessment. General information included socio-demographic variables [permanent residence, gender, age, ethnicity, marital status, degree of education, body mass index (BMI)], lifestyle habits (dietary habits, average sleep duration per night, smoking history, and alcohol history), and past medical history (hypertension, diabetes, coronary heart disease, hearing loss, and visual impairment). Table 1 compiles comprehensive information. All subjects completed the Subjective Cognitive Decline Questionnaire (SCD-Q9) (Hao et al., 2022a, 2022b), the Mini-Mental State Examination (MMSE) (Katzman et al., 1988), and the Montreal Cognitive Assessment (MoCA) (Zhai et al., 2016). Activity of Daily Living (ADL) (Lawton and Brody, 1969; He et al., 1990) scale was utilized to extract information about instrumental daily living activities and physical self-maintenance, such as eating, calling, cooking, handling money, and finishing tasks.

**Table 1:** Independent variable assignment of cognitive function of the study subjects.

Variable	Clarification
Socio-demographic variables	
Permanent residence	Rural area, city
Gender	Male, female
Age (years)	60–69, 70–79, ≥80
Ethnicity	Han, Mongolian
Marital status	Spouse, non-spouse (unmarried, divorced, and widowed)
Degree of education	Primary school and below, middle school, high school and above
Body mass index (BMI)	Light body mass (<18.5 kg/m^2^), normal body mass (18.5–23.9 kg/m^2^), overweight (24.0–27.9 kg/m^2^), and obese (≥28.0 kg/m^2^)
Lifestyle habits	
Dietary habits	Vegetarian-based, meat-based, meat-and-vegetable-based
Average sleep duration per night	<6 h, ≥6 h
Smoking history	At least 1 cigarette per day for more than 1 year
Alcohol history	≥1 drink per week for more than 1 year
Past medical history	
Hypertension	
Diabetes	
Coronary heart disease	
Hearing loss	
Vision loss	

### 2.3 Assessment and diagnosis procedure

Cognitive assessment criteria and methods: all subjects completed the Subjective Cognitive Decline Questionnaire (SCD-Q9), the Mini-Mental State Examination (MMSE), the Montreal Cognitive Assessment (MoCA), and Activity of Daily Living (ADL). It is important to note that these tests provide an overall cognitive profile and do not include specific assessments for each cognitive domain. The ultimate cognitive diagnosis was determined by the expert panel (assistant chief physicians or more senior-level neurologists with over 10 years of experience). Based on the outcome of the above assessment, we finally classified participants into three groups as follows:

1. Normal group: the Normal group was defined as participants who did not have cognitive complaints and achieved a normal score on the administered cognitive screening tests. A total score of <3 on the SCD-Q9.
2. SCD group: using the SCD-I Working group criteria, SCD was identified (Jessen et al., 2014). SCD stands for self-perceived cognitive decline, a decline in daily cognitive functioning. The concept of “normal status” refers to the condition of cognitive functioning that is (subjectively) normal. In contrast, the neuropsychological scales are usually normal. Meanwhile, SCD is persistent and unrelated to an acute incident (Molinuevo et al., 2017). The SCD was not strictly AD preclinical SCD, and the patient did not have PET-CT or cerebrospinal fluid testing. Diagnostic criteria: (1) Subjects reported significant memory loss; (2) Onset within 5 years. (3) Age of onset: 60 years and older. (4) Individuals are concerned about problems related to their cognitive decline. (5) Self-perception that their memory is poorer than that of their peers. (6) Absence of objective clinical impairments of the MCI, with a total score of ≥26 on the MoCA (plus 1 point if degree of education ≤12 years) and a total score of ≥3 on the SCD-Q9.
3. Cognitive impairment group: mild cognitive impairment (MCI): a subset of people with MCI whose impairment is not severe enough to be classified as dementia. The Petersen standard is referenced in the diagnosis of MCI (Petersen, 2004): (1) memory complaints; (2) typical everyday activities; (3) normal general cognitive function; (4) abnormal memory for age; and (5) not dementia. A total score of <26 on the MoCA (plus 1 point if degree of education ≤12 years). Dementia: the fourth edition (DSM-IV) criteria for dementia were utilized to make the diagnosis (Dubois et al., 2007). Dementia was described as a gradual, cumulative process that results in a considerable deterioration from a prior level of functioning in memory and at least one other cognitive domain and is not caused by any other process. The ADL was utilized to evaluate participants whose MMSE scores were <17 or lower in elementary school, <20 in elementary school, and <24 in middle school and higher. If the participants’ ADL score was less than 16, they were deemed to be functionally declining.

### 2.4 Ethical considerations

This investigation has received permission from the Institutional Review Board of Baotou Medical College (No. 2023001). Before the data collection, all respondents were informed of the study’s objectives and methods and their freedom to discontinue participation at any time. Data were only collected from subjects who actively and voluntarily supplied written informed consent to participate in the study.

### 2.5 Data analysis

Version 26.0 of SPSS was used to conduct the statistical analysis. Descriptive statistics were used to describe the samples distributional characteristics. The differences in SCD symptoms among older persons with various sociodemographic traits and lifestyle factors were investigated using chi-square tests and multivariate logistic regression analysis. *p* < 0.05 was the threshold for statistical significance.

## 3 Results

One thousand one hundred twenty participants were included in the analysis. Table 2 describes the characteristics of the participants. There were 206 patients in the normal group, 491 patients in the SCD group, and 423 patients in the cognitive impairment group. Their mean age was 68.48 ± 6.52 years, and 59.3% were women. Most participants were from the city (82.2%) and Han Chinese ethnicity (95.7%). Over half (64.8%) had an degree of educational level above middle school. The mean average sleep duration per night was 6.10 ± 1.48 h. The mean SCDQ9, MMSE, and MoCA scores were 5.24 ± 2.54, 26.42 ± 3.96, and 23.78 ± 5.16, respectively.

**Table 2:** Characteristics compared between the normal, SCD and cognitive impairment group.

		Total sample *N* (%) or mean ± SD	Normal group (*n* = 206)	SCD group (*n* = 491)	Cognitive impairment group (*n* = 423)
Permanent residence					
	Rural area	199(17.8)	10(0.9)	55(4.9)	134(12.0)
	City	921(82.2)	196(17.5)	436(38.9)	289(25.8)
Gender					
	Male	456(40.7)	96(8.6)	195(17.4)	165(14.7)
	Female	664(59.3)	110(9.8)	296(26.4)	258(23.0)
Age (years)		68.48 ± 6.52	67.86 ± 5.86	68.13 ± 6.35	69.19 ± 6.97
Ethnic group					
	Han	1,072(95.7)	198(17.7)	461(41.2)	413(36.9)
	Mongolian	48(4.3)	8(0.7)	30(2.7)	10(0.9)
Marital status					
	Non-spouse	138(12.3)	8(0.7)	57(5.1)	73(6.5)
	Spouse	982(87.7)	198(17.7)	434(38.8)	350(31.3)
Degree of education					
	Primary school and below	394(35.2)	34(3.0)	99(8.8)	261(23.3)
	Middle school	341(30.4)	76(6.8)	165(14.7)	100(8.9)
	High school and above	385(34.4)	96(8.6)	227(20.3)	62(5.5)
BMI					
	Light body mass (<18.5 kg/m^2^)	37(3.3)	4(0.4)	18(1.6)	15(1.3)
	Normal body mass (18.5–23.9 kg/m^2^)	387(34.6)	79(7.1)	151(13.5)	157(14.0)
	Overweight (24.0–27.9 kg/m^2^)	551(49.2)	118(10.5)	258(23.0)	175(15.6)
	Obese (≥28.0 kg/m^2^)	145(12.9)	5(0.4)	64(5.7)	76(6.8)
Dietary habits					
	Vegetarian-based	294(26.3)	28(2.5)	135(12.1)	131(11.7)
	Meat-based	247(22.1)	36(3.2)	91(8.1)	120(10.7)
	Meat-and-vegetable-based	579(51.7)	142(12.7)	265(23.7)	172(15.4)
Average sleep duration per night (hours)		6.10 ± 1.48	6.57 ± 1.49	5.95 ± 1.33	6.05 ± 1.60
Smoking		295(26.3)	33(2.9)	130(11.6)	132(11.8)
Drink		243(21.7)	41(3.7)	121(10.8)	81(7.2)
Hypertension		474(42.3)	84(7.5)	201(17.9)	189(16.9)
Diabetes		196(17.5)	20(1.8)	91(8.1)	85(7.6)
Coronary heart disease		86(7.7)	3(0.3)	32(2.9)	51(4.6)
Hearing Loss		368(32.9)	54(4.8)	149(13.3)	165(14.7)
Visual impairment		572(51.1)	76(6.8)	276(24.6)	220(19.6)
SCDQ9		5.24 ± 2.54	0.62 ± 0.91	6.12 ± 1.12	6.46 ± 1.57
MMSE		26.42 ± 3.96	28.29 ± 2.14	28.27 ± 1.50	23.35 ± 4.65
MoCA		23.78 ± 5.16	26.34 ± 2.96	26.93 ± 1.62	18.88 ± 4.94

Among the 1,120 participants in the analysis, the prevalence of SCD was 43.8%. The study included 195 (39.7%) males and 296 (60.3%) females in SCD group. The SCD group were statistically significant compared to normal group in terms of permanent residence, marital status, degree of education, body mass index (BMI), dietary habits, average sleep duration per night, smoking, diabetes, coronary artery disease and history of previous visual impairment (*p* < 0.05). Table 3 compiles comprehensive information.

**Table 3:** Characteristics compared between the normal and SCD group.

		Number of Investigators (*n* = 697)	Cognitive function *n* (%)			
			Normal group (*n* = 206)	SCD group (*n* = 491)	χ^2^	*P*
Permanent residence					6.914	0.009
	Rural area	65(9.3)	10(4.9)	55(11.2)		
	City	632(90.7)	196(95.1)	436(88.8)		
Gender					2.830	0.093
	Male	291(41.8)	96(46.6)	195(39.7)		
	Female	406(58.2)	110(53.4)	296(60.3)		
Age (year)					2.340	0.310
	60–69	425(61.0)	121(58.7)	304(61.9)		
	70–79	232(33.3)	76(36.9)	156(31.8)		
	≥80	40(5.7)	9(4.4)	31(6.3)		
Ethnic group					1.396	0.237
	Han	659(94.5)	198(96.1)	461(93.9)		
	Mongolian	38(5.5)	8(3.9)	30(6.1)		
Marital status					10.242	<0.001
	Non-spouse	65(9.3)	8(3.9)	57(11.6)		
	Spouse	632(90.7)	198(96.1)	434(88.4)		
Degree of education					1.476	0.478
	Primary school and below	133(19.1)	34(16.5)	99(20.2)		
	Middle school	241(34.6)	76(36.9)	165(33.6)		
	High school and above	323(46.3)	96(46.6)	227(46.2)		
BMI					21.001	<0.001
	Light body mass (<18.5 kg/m2)	22(3.2)	4(1.9)	18(3.7)		
	Normal body mass (18.5–23.9 kg/m2)	230(33.0)	79(38.3)	151(30.8)		
	Overweight (24.0–27.9 kg/m2)	376(53.9)	118(57.3)	258(52.5)		
	Obese (≥28.0 kg/m2)	69(9.9)	5(2.4)	64(13.0)		
Living habits						
Dietary habits					17.645	<0.001
	Vegetarian-based	163(23.4)	28(13.6)	135(27.5)		
	Meat-based	127(18.2)	36(17.5)	91(18.5)		
	Meat-and-vegetable-based	407(58.4)	142(68.9)	265(54.0)		
Average sleep duration per night					8.762	0.003
	<6 h	222(31.9)	49(23.8)	173(35.2)		
	≥6 h	475(68.1)	157(76.2)	318(64.8)		
Smoking					8.857	0.003
	No	534(76.6)	173(84.0)	361(73.5)		
	Yes	163(23.4)	33(16.0)	130(26.5)		
Drink					1.828	0.176
	No	535(76.8)	165(80.1)	370(75.4)		
	Yes	162(23.2)	41(19.9)	121(24.6)		
Past history						
Hypertension					0.002	0.969
	No	412(59.1)	122(59.2)	290(59.1)		
	Yes	285(40.9)	84(40.8)	201(40.9)		
Diabetes					8.441	0.004
	No	586(84.1)	186(90.3)	400(81.5)		
	Yes	111(15.9)	20(9.7)	91(18.5)		
Coronary heart disease					7.793	0.005
	No	662(95.0)	203(98.5)	459(93.5)		
	Yes	35(5.0)	3(1.5)	32(6.5)		
Hearing Loss					1.201	0.273
	No	494(70.9)	152(73.8)	342(69.7)		
	Yes	203(29.1)	54(26.2)	149(30.3)		
Visual impairment					21.666	<0.001
	No	345(49.5)	130(63.1)	215(43.8)		
	Yes	352(50.5)	76(36.9)	276(56.2)		

Table 4 shows the results of multivariable logistic regression analysis. Compared with those with normal weight, individuals with obesity had a higher risk of SCD (OR = 6.159, 95% CI: 2.303–16.473, *p* < 0.001). Compared with those with a normal diet, individuals with a vegetarian diet had a higher risk of SCD (OR = 2.961, 95% CI: 1.820–4.818, *p* < 0.001). Compared with those who do not smoke, individuals who have smoked for a long time had a higher risk of SCD (OR = 1.919, 95%CI:1.189–3.096, *p* < 0.05). People with diabetes and coronary heart disease, respectively, have a higher risk of SCD (OR = 1.958: 95%CI, 1.132–3.387, *p* < 0.05; OR = 3.906, 95%CI: 1.136–13.433, *p* < 0.05, respectively). Those With Visual impairment had a high risk of SCD (OR = 1.855, 95%CI: 1.288–2.673, *p* = 0.001). Compared with no spouse, individuals with a spouse had a lower risk of SCD (OR = 0.408, 95%CI: 0.182–0.915, *p* < 0.05). Compared with those having an Average sleep duration per night <6 h, individuals with an Average sleep duration per night >6 h had a lower risk of SCD (OR = 0.585, 95%CI:0.388–0.883, *p* < 0.05).

**Table 4:** Multivariable logistic regression analysis of SCD prevalence.

Explanatory variables	*β*	S.E.	Wald *χ*^2^	OR	*P*	95% CI of OR value	
						Lower limit	Upper limit
Permanent residence	−0.592	0.383	2.387	0.122	0.553	0.261	1.172
Marital status	−0.896	0.412	4.729	0.030	0.408	0.182	0.915
BMI			14.421	0.002			
BMI (1)	0.816	0.595	1.882	0.170	2.260	0.705	7.249
BMI (2)	0.137	0.193	0.501	0.479	1.147	0.785	1.676
BMI (3)	1.818	0.502	13.118	0.000	6.159	2.303	16.473
Dietary habits			19.871	0.000			
Dietary habits (1)	1.086	0.248	19.111	0.000	2.961	1.820	4.818
Dietary habits (2)	−0.007	0.253	0.001	0.979	0.993	0.605	1.629
Average sleep duration per night	−0.535	0.210	6.531	0.011	0.585	0.388	0.883
Smoking	0.652	0.244	7.126	0.008	1.919	1.189	3.096
Diabetes	0.672	0.280	5.774	0.016	1.958	1.132	3.387
Coronary heart disease	1.363	0.630	4.674	0.031	3.906	1.136	13.433
Visual impairment	0.618	0.186	11.020	0.001	1.855	1.288	2.673

For binary and hierarchical variables, the lower assigned value is used as a reference; Reorder BMI with “4 = normal weight” as a reference. BMI (1) refers to “1 = underweight vs. normal weight,” BMI (2) refers to “2 = overweight vs. normal weight,” and BMI (3) refers to “3 = obese vs. normal weight”. The type of vegetarian diet is based on the reference of “3 = normal diet.” The type of vegetarian diet (1) refers to “vegetarian vs. normal diet,” and the type of vegetarian diet (2) refers to “meat vs. normal diet”.

Table 5 shows the subgroup analysis of the SCD group regarding gender (male or female). Our study showed that the SCD population of males and females is 195 (39.7%) and 296 (60.3%), respectively, and the majority of respondents were between ages 60 and 69 (61.9%). Based on the gender analysis, our results revealed that the difference in marital status was statistically significant (*p* < 0.001). In the spouse population, the prevalence in females (50.5%) was notably higher than in males (39.9%). Based on the gender analysis, the difference in dietary habits was statistically significant (*p* < 0.001), and the majority of participants were meat-and-vegetable-based (54.0%). The difference in average sleep duration per night<6 h and ≥6 h was statistically significant (*p* < 0.001), which is ≥6 h the prevalence of in females (34.4%) was higher than males (30.3%), and in <6 h the prevalence of in females (25.8%) was notably higher than males (9.4%). The difference in smoking and drinking was statistically significant (*p* < 0.001), and the majority of the population were females with no smoking (58.5%) and drinking (58.0%).

**Table 5:** The subgroup analysis of SCD group.

		Gender			
		Male *N* = 195	Female *N* = 296	*χ* ^2^	*P*
Permanent residence				0.114	0.735
	Rural area	23(11.8)	32(10.8)		
	City	172(88.2)	264(89.2)		
Age (years)				11.473	0.003
	60–69	104(53.3)	200(67.6)		
	70–79	73(37.4)	83(28.0)		
	≥80	18(9.2)	13(4.4)		
Marital status				15.418	<0.001
	Non-spouse	9(4.6)	48(16.2)		
	Spouse	186(95.4)	248(83.8)		
Degree of education				4.366	0.113
	High school and above	99(50.8)	128(43.2)		
	Middle school	65(33.3)	100(33.8)		
	Primary school and below	31(15.9)	68(23.0)		
BMI				3.247	0.355
	Light body mass (<18.5 kg/m^2^)	8(4.1)	10(3.4)		
	Normal body mass (18.5–23.9 kg/m^2^)	57(29.2)	94(31.8)		
	Overweight (24.0–27.9 kg/m^2^)	110(56.4)	148(50.0)		
	Obese (≥28.0 kg/m^2^)	20(10.3)	44(14.9)		
Dietary habits				24.241	<0.001
	Vegetarian-based	36(18.5)	99(33.4)		
	Meat-based	54(27.7)	37(12.5)		
	Meat-and-vegetable-based	105(53.8)	160(54.1)		
Average sleep duration per night				19.220	<0.001
	<6 h	46(23.6)	127(42.9)		
	≥6 h	149(76.4)	169(57.1)		
Smoking				210.290	<0.001
	Yes	121(62.1)	9(3.0)		
	No	74(37.9)	287(97.0)		
Drink				175.770	<0.001
	Yes	110(56.4)	11(3.7)		
	No	85(43.6)	285(96.3)		
Hypertension				11.181	0.001
	Yes	62(31.8)	139(47.0)		
	No	133(68.2)	157(53.0)		
Diabetes				2.651	0.103
	Yes	43(22.1)	48(16.2)		
	No	152(77.9)	248(83.8)		
Coronary heart disease				1.920	0.166
	Yes	9(4.6)	23(7.8)		
	No	186(95.4)	273(92.2)		
Hearing loss				0.321	0.571
	Yes	62(31.8)	87(29.4)		
	No	133(68.2)	209(70.6)		
Visual impairment				0.665	0.415
	Yes	114(58.5)	162(54.7)		
	No	81(41.5)	134(45.3)		

## 4 Discussion

For older persons to continue living independently, cognitive function is essential. People with and without cognitive impairment can have SCD, which can negatively impact their day-to-day functioning. Thus, this study looked at variables related to SCD and cognitive performance in Baotou. The current research contributes new evidence to the body of knowledge that may be utilized to create treatments for older individuals.

To the best of our knowledge, some research has examined the prevalence of SCD; however, studies have yet to be conducted in the North of China. This cross-sectional study is based on a population aged 60 years or older, analyzing the prevalence and risk factors of SCD in 491 older adults. Our study showed a prevalence of SCD of 43.8%, which is higher than America (25.5%) and Australia (36.7%) (Brodaty et al., 2017; Liew, 2019), and is similar to other studies in China (40.07–42.0%) (Ruan et al., 2020; Wen et al., 2021; Xu et al., 2023). In the cohort studies, Liew (2019) focused on people over 50 in the United States, whereas Brodaty et al. (2017) examined the aging population of 70–90 in Australia. Given the variations in the age range, geographical locations, and methodologies of the study population, heterogeneity would be anticipated among these investigations. The lack of defined operational procedures for assessing SCD makes comparing prevalence rates across research challenging, even though the SCD-I Working Group has presented a consensual definition of pre-MCI SCD.

The main finding of the study was that the prevalence of SCD in females (60.3%) is higher than in males (39.7%). There are differences between males and females when it comes to mild cognitive impairment and Alzheimer’s disease (Podcasy and Epperson, 2016). Alzheimer’s disease is more common in females than in males (Brown and Patterson, 2020), yet some research suggests that this difference is more likely to be explained by age than by gender because females often live longer than males (Hebert et al., 2001). On the contrary, the study on the connection between gender and SCD is contradictory. According to research by Wang and Tian (2018) on memory tasks, females with SCD fared noticeably better than males with SCD. The absence of consistent findings regarding gender differences in SCD highlights the need for more study in this field.

Biologically, factors such as BMI, dietary habits, sleep duration, smoking, and chronic conditions like diabetes, visual impairment, and coronary heart disease play a crucial role in SCD. Certain risk factors, such as diabetes and coronary heart disease, have been previously reported in the Chinese population (Schliep et al., 2022). In line with a cross-sectional study conducted among Chinese citizens 60 or older (Luo et al., 2024), we discovered that older persons with visual impairments have a higher chance of acquiring SCD. Visual impairment affects the activities in which older persons participate, affecting cognitive performance (Zheng et al., 2018). This may be due to impairments in cognitive functioning as a result of reduced visual acuity, fewer external stimuli related to cognitive activity, and increased risks such as social isolation (Bikbov et al., 2022). Maintaining healthy vision may be a crucial intervention method for minimizing cognitive alterations, as there is a correlation between cognitive performance and vision. Therefore, to avoid visual impairment and SCD, it is recommended that older adults follow a healthy lifestyle and have regular eye exams. Multivariable logistic regression analysis showed that smoking was an independent risk factor for SCD in older adults. This is consistent with the results of a study in Canada that showed an increased risk of SCD in older adults who smoked for a long time (Hopper et al., 2023). Research suggests that increased levels of oxidative stress and free radicals are linked to cognitive disorders in long-term smokers (Amini et al., 2021). Smoking can hasten the aging process’s impact on cognitive function. However, others researchers thought that because smoking had short-term effects on the cholinergic system, it protects working memory and executive function (Swan and Lessov-Schlaggar, 2007). Hence, there is a need to stop smoking.

Lifestyle factors were associated with an increased risk for SCD. It has been shown that long-term vegetarians are more prone to subjective cognitive decline than those who consume a normal diet. This may be because animal organs and meat are rich in folate, and folate deficiency can cause cognitive impairment. A considerable amount of evidence shows that folate deficiency is associated with an increased risk of cognitive decline (Jiang et al., 2023). Hence, it is vital for the elderly to balance a reasonable meat and vegetable diet and moderate folic acid supplementation, which could prevent the occurrence of SCD and cognitive impairment. Additionally, our research revealed a connection between obesity and SCD. Few pertinent longitudinal studies have looked at the relationship between obesity and the advancement of SCD in older persons who are cognitively normal. A high BMI in midlife was linked to late-life cognitive decline and dementia, according to a prior meta-analysis. In contrast, a high BMI in late life seemed to have protective benefits against dementia (Qu et al., 2020). Specific fat components may affect cognitive decline and dementia differently and are linked to diverse metabolic profiles (Carr et al., 2004). Obesity and cognitive decline are linked by a multitude of intricate pathogenic mechanisms, such as insulin resistance, chronic inflammation, and blood–brain barrier disruption (Barber et al., 2021). This could eventually hasten the onset of cognitive decline and raise the possibility of developing Alzheimer’s disease (AD). Therefore, seniors are encouraged to participate in moderate physical activities, such as walking, dancing, playing ball, and swimming, and maintain an appropriate and reasonable BMI, which can prevent the onset of memory loss and subjective cognitive decline.

Finally, evidence suggests that sleep is closely related to cognitive performance and brain health (Xu et al., 2020). In our findings, the prevalence of SCD in people who sleep more than 6 h per night was lower than that of those who sleep less than 6 h per night, suggesting that the SCD prevention potential was related to sleep. The outcome is in line with a recent study in five Nordic cohorts of older adults, which indicates that insomnia symptoms and less sleep duration resulted in worse and steeper cognitive decline (Overton et al., 2024). A population-based survey from all ages of adults found that self-reported sleep problems were linked to accelerated cognitive deterioration (Köhler et al., 2023). However, other research has confirmed the u-shape theory of sleep duration and cognition, which holds that short and long sleep are associated with cognitive decline (Kronholm et al., 2009; Mohlenhoff et al., 2018). A pooled cohort study found that people who slept for longer than 10 h or for 5 h as opposed to 7 h per night had inferior global cognition; however, this difference did not hold for people who slept for 6, 8, or 9 h (Ma et al., 2020). Therefore, promoting sleep quality and appropriately extending sleep length can help avoid sleep-related cognitive impairments while promoting healthy brain aging throughout the adult lifespan.

In this study, age affected subjective cognitive impairment in older adults. Previous research has shown that age and educational attainment are linked to cognitive function, but SCD was not connected to either of these factors (Dale et al., 2018; Min, 2018). On the contrary, cognitive function was not associated with age and educational level, but SCD with age in our study, probably because of the geography of the Baotou area and the fact that part of the population is from rural areas with low literacy. Frailty, a geriatric syndrome, is characterized by an increased susceptibility to stressors because of a decreased homeostatic reserve brought on by an age-related multisystem physiological change that has been associated with an increased risk of cognitive impairment (Palermo, 2020). Regarding public health and clinical practice, frailty is the most problematic manifestation of population aging (Karanth et al., 2024). Older adults may have physical frailty and cognitive frailty and are more likely to suffer from cognitive decline and memory decline (Zhang et al., 2024). Hence, assessing frailty in the elderly may identify those with SCD and cognitive impairment.

The overall prevalence of SCD in the rural population was higher than that in the urban population, suggesting the dementia prevention potential in rural areas is greater than in urban areas. In cross-sectional analysis, we found that people living in rural areas were independently associated with SCD. This may be because people living in rural areas are less educated, often engage in physical activities, and spend less time performing brain activities. This is consistent with the finding of a systematic review that people who are physically active for long periods are at greater risk for cognitive impairment than those in intellectually demanding occupations (Gracia Rebled et al., 2016). There was a strong correlation between cognitive function and cognitive activity (Lee et al., 2020). Another reason could be that individuals with lower social/emotional support (SES) who live in rural areas tend to have fewer healthy relationships and participate in fewer social activities, which lowers brain stimulation and increases depression, eventually leading to the development of SCD. Conversely, individuals with SES who live in cities are more likely to participate in social activities and have better brain functioning (Weng et al., 2020). So, appropriate cognitive activity is essential for maintaining cardiovascular health and may protect against cognitive deterioration.

Socially, variables such as marital status significantly impact cognitive health. Our subgroup analysis showed that non-spouse people were associated with a higher risk of subjective cognitive decline, consistent with the results of a community-based prospective study (Cheng et al., 2023). Divorce may be related to a reduced social network, fewer mentally stimulating activities, or an increased risk of depression, all of which may have a role in the development of SCD (Weng et al., 2020). Elderly singles are more likely to experience loneliness and a sense of social isolation. Significantly, loneliness affects both cognitive and physical health and frequently results in cognitive decline and the development of neurocognitive diseases (Luchetti et al., 2020). Psychologically, cognitive decline may contribute to loneliness in older adults by impeding social engagement with friends and family and making it more challenging to assess relationship satisfaction (Morese and Palermo, 2022). Perceived social isolation is associated with a higher risk of lower overall cognitive performance, a faster rate of cognitive decline, and poorer executive functioning (Cacioppo and Hawkley, 2009). Previous studies have shown that social isolation is an independent risk factor in dementia (Livingston et al., 2020). This research implies that social network factors might be the first focus of SCD preventive measures. In order to promote a positive outlook and lead meaningful lives, seniors are therefore encouraged to engage in social and leisure activities.

The high incidence of SCD emphasizes the significance of identifying risk factors and implementing targeted interventions to prevent it from progressing to MCI, AD, and other types of dementia. The elimination of the following risk factors could potentially prevent 40% of dementia cases, according to a 2020 study by the Lancet Commission on Dementia Prevention, Intervention, and Care: low social contact, smoking, obesity, diabetes, hearing impairment, hypertension, less education, physical inactivity, depression, excessive alcohol consumption, traumatic brain injury, and air pollution (Livingston et al., 2020). As a result, integrating the study findings with the research guidelines yields evidence-based suggestions for preventing SCD and cognitive impairment. These interventions may also serve as viable preventative treatments to delay age-related cognitive decline and neurodegeneration.

Sociodemographic characteristics and way of living were strongly associated with SCD symptoms in our study. This study concludes by identifying important variables affecting the subjective cognitive ability of older adults. It is imperative to take proactive measures to manage these modifiable risk factors to postpone the development of more severe cognitive deficits. Furthermore, more research is required to determine whether environmental or physiological factors cause gender disparities in SCD. Future studies may also need to examine the connection between SCD and social determinants of health.

## 5 Strengths and limitations

This study has several advantages. First, this study is the first to screen the prevalence of SCD and analyze related risk factors in the elderly population over 60 in Inner Mongolia and to conduct health degree of education and standardized management of the disease. Second, it is the first study to investigate the cognitive level of a large sample of the elderly population in Inner Mongolia, which fills the gap in the epidemiological data of cognitive disorders and has great significance for preventing and managing dementia chronic diseases. However, there are some limitations to this study. Firstly, much like in previous cross-sectional research, it is not possible to prove a causality between SCD and sociodemographic variables and chronic illnesses. Secondly, even though all models included gender as a covariate, it’s likely that our sample is not typical of the general population. Thirdly, we limited our study to those over 60 years of age and the connection of SCD symptoms. Fourthly, our study were limited to a single Chinese city district, making it impossible for them to accurately represent all northern Chinese.

## 6 Conclusion

In summary, the prevalence of SCD in the Baotou area is 43.8% in older adults over 60 years of age, and there are multiple risk factors associated with SCD, including living in rural areas, having no spouse, obesity, smoking, diabetes mellitus, coronary heart disease, and visual impairment. Dietary habits, average sleep duration per night, and smoking are essential factors between men and women contributing to subjective cognitive decline. SCD can now be prevented by early detection and early intervention of controllable risk factors, and this study has important implications for the prevention of mild cognitive impairment and Alzheimer’s disease.

## Data Availability

The raw data supporting the conclusions of this article will be made available by the authors, without undue reservation.
